# Putative Bidirectionality of Chronic Obstructive Pulmonary Disease and Periodontal Disease: A Review of the Literature

**DOI:** 10.3390/jcm12185935

**Published:** 2023-09-13

**Authors:** Hiroyuki Tamiya, Akihisa Mitani, Masanobu Abe, Takahide Nagase

**Affiliations:** 1 Division for Health Service Promotion, The University of Tokyo, 7-3-1 Hongo, Bunkyo-ku, Tokyo 113-0033, Japan; 2 The Department of Respiratory Medicine, The University of Tokyo Hospital, 7-3-1 Hongo, Bunkyo-ku, Tokyo 113-8655, Japan; 3 Department of Sensory and Motor System Medicine, Graduate School of Medicine, The University of Tokyo, Tokyo 113-8654, Japan

**Keywords:** oral health, periodontal disease, chronic obstructive pulmonary disease, smoking, exacerbation, quality of life, pulmonary function

## Abstract

The prevalence of chronic obstructive pulmonary disease (COPD) is increasing worldwide and is currently the third leading cause of death globally. The long-term inhalation of toxic substances, mainly cigarette smoke, deteriorates pulmonary function over time, resulting in the development of COPD in adulthood. Periodontal disease is an inflammatory condition that affects most adults and is caused by the bacteria within dental plaque. These bacteria dissolve the gums around the teeth and the bone that supports them, ultimately leading to tooth loss. Periodontal disease and COPD share common risk factors, such as aging and smoking. Other similarities include local chronic inflammation and links with the onset and progression of systemic diseases such as ischemic heart disease and diabetes mellitus. Understanding whether interventions for periodontal disease improve the disease trajectory of COPD (and vice versa) is important, given our rapidly aging society. This review focuses on the putative relationship between COPD and periodontal disease while exploring current evidence and future research directions.

## 1. Introduction

Chronic obstructive pulmonary disease (COPD) is an inflammatory disease of the lungs caused by long-term inhalation of toxic substances, mainly tobacco smoke. COPD is a lifestyle-related disease that develops in middle-aged and older adults [1]. COPD is characterized by airflow limitation. Specifically, in persons with COPD, the ratio of forced expiratory volume in 1 s (FEV_1_) to forced vital capacity (FVC) is <0.7; the predicted value of FEV_1_/FVC varies slightly with age and ethnicity but is generally around 0.8 [2]. The major symptoms of COPD include coughing, sputum expectoration, breathlessness on exertion, and lower respiratory tract infections that occur more frequently or last longer than expected [3]. COPD is estimated to affect 328 million people worldwide and is the third leading cause of death globally [4,5]. In addition, moderate-to-severe COPD imposes a substantial economic burden on healthcare providers [6].

Periodontal disease involves chronic inflammation of the tooth-supporting tissues, including the gingiva, periodontal ligament, and alveolar bone. Over time, this inflammation leads to tooth loss and bone damage [7,8]. Previous studies found that 50–90% of adults worldwide demonstrate signs of periodontal disease [9,10], with severe disease evident in 9.8% of adults globally [11]. Periodontal disease compromises oral hygiene and triggers systemic diseases that may produce significant health burdens and increase the risk of all-cause and disease-specific mortality from cardio- and cerebrovascular diseases [12]. Given our rapidly aging society, and that periodontal disease affects most adults over 50, effective management is important [13].

There is growing interest in the interaction between periodontal disease and respiratory disease—including COPD. Periodontal disease and COPD share several risk factors, such as smoking, age, obesity, socioeconomic status, and living conditions [14]. Moreover, given the connections between the upper and lower airways, poor oral health may exert deleterious effects on the human respiratory tract. Periodontal disease and COPD induce systemic inflammation and can affect remote organs [7,15]. The hypothesis that COPD and periodontal disease are causally related and that treating one might affect the severity and progression of the other is supported, to some extent, by the available evidence [16]. However, Riley et al. found that healthcare providers did not always understand the relationship between oral health and COPD [17]. This review will discuss the relationship between the two diseases and suggest future research directions.

## 2. The Relationship between Periodontal Disease and COPD

Numerous studies have been conducted to examine the association between periodontal disease or poor periodontal hygiene and the development of COPD, and a number of systematic reviews and meta-analyses integrating these studies have been reported. Furthermore, previous reports suggest that periodontal disease may also affect various outcomes that may be related to the prognosis of COPD, such as pulmonary function, quality of life (QOL), and exacerbation frequency [8,18]. Herein, we review published studies that examined the association between periodontal disease and COPD.

### 2.1. The Association between Periodontal Disease and the Development of COPD

A systematic review of three studies by Azarpazhooh et al. found an association (odds ratio [OR] < 2.0) between periodontal disease and COPD [19].

A meta-analysis by Garcia et al. found that worsening periodontal health was significantly associated with an elevated risk of COPD (chronic bronchitis and emphysema), with ORs ranging from 1.45–4.50. Meanwhile, the authors speculated that cigarette smoking may have confounded the results because periodontal status contributed to an increased risk of COPD in current smokers (relative risk [RR] = 1.63, 95% confidence interval [CI] = 1.20–2.21), but there was no such association in never-smokers (RR = 1.19, 95% CI = 0.71–1.98) [20]. In a meta-analysis of 14 studies, Shi et al. observed that patients with COPD (*n* = 3348) had poorer periodontal health than a non-COPD cohort (*n* = 20,612); namely, deeper periodontal pockets, greater clinical attachment loss (CAL), poorer oral hygiene, more gingival tissue inflammation and bleeding, and fewer remaining teeth [21]. However, the limited information did not allow them to examine the impact of common risk factors for the two diseases on the overall effect. Zeng et al. also conducted a meta-analysis of 14 observational studies (3988 patients with COPD and 22,871 control participants) and found a significant association between probing pocket depth (PD) and COPD (OR = 2.08, 95% CI = 1.48–2.91), although they detected moderate publication bias with an Egger linear regression test (OR = 3.43, 95% CI = 1.76–5.10; *p* < 0.001) [22]. According to the meta-analysis by Gomes-Filho et al., which included five studies, periodontitis was significantly associated with COPD (adjusted OR = 1.78, 95% CI = 1.04–3.05), and the I^2^ was 37.9% (95% CI: 0–79%), indicating moderate between-study variability [23]. They also pointed out that the diagnostic definitions of periodontal disease used in the studies varied. Wu et al. found a significant association between various respiratory diseases, including COPD (adjusted OR = 1.64, 95% CI = 1.19–2.27, *p* < 0.05, I^2^ = 42.3%), and periodontal health in their meta-analysis. For example, the following oral hygiene parameters were worse in the COPD than in the non-COPD group: Oral Hygiene Index (OHI), number of remaining teeth, alveolar bone loss (ABL), PD, CAL, plaque index (PI), and gingival index (GI) [24]. Molina et al. analyzed 12 studies and found a significant association between periodontal disease and COPD, with an adjusted OR of 1.28 and 95% CI = 1.16–1.42 (*p* < 0.001, I^2^ = 30.5). In addition, the FEV_1_/FVC ratio was 4.94% lower in patients with periodontitis [25].

Collectively, these results suggest that there may be an association between periodontal disease and COPD; however, the magnitude of the association appears to be small in some of the studies. There was also moderate heterogeneity among studies in some meta-analyses. Periodontitis and COPD share common risk factors, e.g., smoking, aging, etc. Thus, adequate control of these covariates is of paramount importance when assessing the association between diseases. Variations in the diagnostic definition of periodontal disease must also be considered. Most of the studies analyzed were cross-sectional or case-control studies; however, prospective investigations with rigorous diagnostic criteria would be needed to better clarify the association.

### 2.2. Does Periodontal Disease Promote a Decline in Pulmonary Function?

Several studies have explored the impact of periodontal disease on the decline of pulmonary function (Table 1).

Pérez Barrionuevo et al. investigated the association between the community periodontal index (CPI) and pulmonary function in two cohorts of young and middle-aged Norwegian adults. Participants with a CPI of 3–4 (worse periodontal health) had significantly lower FEV_1_/FVC ratios than those with a CPI of 0 (healthy periodontal status), although this study was not limited to patients with COPD [26].

Holtfreter et al. examined the potential associations between periodontal variables and pulmonary function in a cross-sectional study of 1463 participants in Germany [27]. The mean CAL was significantly associated with reduced dynamic lung volume (FVC and FEV_1_), static lung volume (functional residual capacity, ascertained via body plethysmography), and airflow limitation (expressed by FEV_1_/FVC ratio, maximal expiratory flow at 25% of FVC [MEF25%], and residual volume/total lung capacity ratio).

According to a cross-sectional study among 826 older Caucasian dentate individuals, radiographic periodontal bone loss was independently associated with airflow limitation (FEV_1_/FVC ratio < 0.7), with an OR of 2.31 [28]. Katancik et al. examined the association between periodontal disease and airflow limitation in 860 community-dwelling, high-functioning older adults. Participants with airflow limitation had significantly worse GI and loss of attachment values than those with normal pulmonary function. After stratifying by smoking status and adjusting for covariates, a significant association was found between periodontal disease and airflow limitation among former smokers [29].

Si et al. stratified Chinese patients with COPD by disease severity according to their body mass index, airflow obstruction, dyspnea, and exercise capacity (BODE) index, and investigated periodontal parameters including PD, attachment loss, PI, alveolar bone volume, and the number of teeth in each group. All parameters were significantly associated with all stages of COPD, and participants with more severe COPD were more likely to have more severe periodontal disease. In addition, individuals with higher BODE scores experienced significantly higher attachment loss, bleeding index (BI), PI, and ABL. Additionally, those with COPD had significantly fewer teeth than the control group (BODE = 0) [30].

Moeintaghavi et al. [31] and Peter et al. [32] found significant negative correlations between FEV_1_ values and PI and other attachment loss variables.

A nested case-control study that used the Veterans Affairs Dental Longitudinal Study cohort and included a 25-year follow-up period found that radiographic ABL status at baseline was an independent risk factor for COPD after adjusting for certain risk factors, including smoking [33]. However, this study defined COPD as an FEV_1_ < 65% of the predicted value; this differs from the common diagnostic criteria for COPD.

Tan et al. reported a significant relationship between the Oral Hygiene Index—Simplified (OHI-S), CAL, and the percentage predicted FEV_1_ values when they compared patients with COPD and the controls. They identified a significant negative correlation between the relative content of *Porphyromonas gingivalis*—a major periodontal disease pathogen—and the percentage predicted FEV_1_ in patients with COPD. They additionally found that patients with COPD were more likely to carry *P*. *gingivalis*, *Klebsiella pneumoniae*, *Pseudomonas aeruginosa*, and *Streptococcus pneumoniae* than the controls. The authors speculated that oral plaque might have acted as a reservoir for pathogenic respiratory bacteria [34].

A population-based cohort study in Ireland uncovered a significant association between chronic periodontitis (as measured via mean CAL) and reduced pulmonary function (as measured via percentage predicted FEV_1_). For example, doubling the mean CAL corresponds to a −3.33% change in predicted FEV_1_ after adjusting for potential confounding variables [35].

A case-control study in Iran showed that PD, bleeding on probing (BOP), and CAL were negatively correlated with the percentage predicted values of FEV_1_ and FEV_1_/FVC ratios. The authors also found that PD, BOP, and CAL were positively correlated with COPD Assessment Test scores (where higher scores indicate the deterioration of COPD symptoms) [36].

Lee et al. used data from the National Health and Nutrition Examination Survey (NHANES) III (1988–1994) to investigate the relationship between pulmonary function and periodontitis. Their study, which included 10,645 participants, demonstrated that percentage predicted FEV_1_, percentage predicted FVC, and FEV_1_/FVC values decreased gradually as periodontitis severity increased. Both obstructive and restrictive spirometry patterns were significantly associated with periodontitis severity [37].

Chen et al. performed a similar study using the NHANES 2009–2012 database. Their examination of 6313 adults revealed significantly increased adjusted ORs for airflow obstruction in patients with moderate and severe periodontitis compared to those without periodontitis: 1.38 and 1.47, respectively. Mean attachment loss and mean PD were negatively correlated with FEV_1_, FVC, and FEV_1_/FVC, regardless of smoking history. A dose–response relationship was demonstrated, with an increased risk of airflow obstruction as the mean attachment loss and mean PD increased [38].

Hämäläinen et al. conducted a 5-year prospective cohort study of dental status and pulmonary function in community-dwelling older adults in Finland. Male participants with complete dentures had the lowest FEV_1_ at the baseline. After 5 years, FEV_1_ values declined the most in participants with poor periodontal status or complete dentures (−9.4%); however, no declines were observed among those without periodontal disease (1.0%). The authors speculate this may be because periodontal disease and complete dentures act as reservoirs for harmful pathogens, potentially contributing to aging-related reductions in FEV_1_ [39]. Although the association between periodontitis and restrictive (adjusted OR, 1.059) or obstructive pulmonary impairment (adjusted OR, 1.140) did not reach statistical significance in a study using Korean NHANES data [40], the results from other studies suggest that periodontal disease may be connected to impaired pulmonary function.

In contrast, some reports suggest that smoking and aging may have a substantial effect. For example, Henke et al. investigated the association between pulmonary function, including several indicators of peripheral airway involvement and various oral hygiene parameters. They observed that dentures, missing teeth, oral mucosal disease, and a higher index value for decayed, missing, and filled permanent teeth (DMFT) were associated with lower MEF25%. However, only dentures were associated with a lower MEF25% percentage predicted and FEV_1_/FVC in the adjusted logistic regression analysis. Because periodontitis and DMFT were associated only with age and the amount of smoking, they concluded that much of the association between pulmonary function and poor oral hygiene could be explained by smoking and age [41].

**Table 1:** Studies examining the relationship between periodontal disease and pulmonary function.

Author (Year)	Location	Study Design	Study Population	*n*	Measured Outcome	Main Findings
Pérez Barrionuevo et al., 2018 [26]	Norway	Cross-sectional	Norwegian participants in the Respiratory Health in northern Europe, Spain, and Australia (RHINESSA) generation study and the third wave of the European Community Respiratory Health Survey (ECRHS) study	656	Periodontal parameter: CPI Pulmonary function measurement: spirometry	Participants with CPI 3–4 (worse periodontal health) had significantly lower FEV_1_/FVC ratios compared to participants with CPI 0 (healthy periodontal status): regression coefficients β (95% CI) = −0.032 (−0.055, −0.009), *p*-trend = 0.004.
Holtfreter et al., 2013 [27]	Germany	Cross-sectional	Participants in the Study of Health in Pomerania	1463	Periodontal parameters: CAL, PD, and number of missing teeth Pulmonary function measurements: spirometry, body plethysmography, and diffusing capacity of the lung for carbon monoxide	Mean CAL was significantly associated with reduced FVC (*p* < 0.05), FEV_1_ (*p* < 0.001), functional residual capacity (*p* < 0.001), FEV_1_/FVC ratio (*p* < 0.01), maximal expiratory flow at 25% of FVC (*p* < 0.05), and residual volume/total lung capacity ratio (*p* < 0.001).
Winning et al., 2020 [28]	Sweden	Cross-sectional	Individuals selected from the Swedish civil registration database representing the aging population in Karlskrona, Sweden	826	Periodontal parameter: periodontal bone loss Pulmonary function measurement: spirometry	The percentage of participants in the airflow limitation (FEV_1_/FVC < 0.7) group who presented with periodontitis was 65.1%, compared to 41.5% in the group with normal pulmonary function (*p* < 0.001). Periodontitis was independently associated with airflow limitation (OR = 2.31).
Katancik et al., 2005 [29]	USA	Cross-sectional	Community-dwelling, well-functioning older adults selected from participants enrolled in the Health, Aging, and Body Composition Study (Health ABC)	860	Periodontal parameters: PI, GI, PD, and LOA Pulmonary function measurement: spirometry	Participants with airflow limitation (defined as a reduced FEV_1_/FVC as determined by age-, gender-, and race-normalized values) had significantly worse GI (*p* = 0.022) and LOA (*p* = 0.009) than those with normal pulmonary function.
Moeintaghavi et al., 2018 [31]	Iran	Cross-sectional	Patients with COPD who had been referred to the specialty clinic	50	Periodontal parameters: PD, LOA, GI, and PI Pulmonary function measurements: spirometry and SpO_2_	The FEV_1_ and FVC indices showed significant negative correlations with PI (FEV_1_: *r* = −0.481, *p* < 0.001; FVC: *r* = −0.296, *p* = 0.037) and LOA (FEV_1_: *r* = −0.370, *p* = 0.008; FVC: *r* = −0.370, *p* = 0.008). The SpO_2_ index showed a significant negative correlation with GI (*r* = −0.339, *p* = 0.016) and attachment loss (*r* = −0.319, *p* = 0.024) variables.
Winning et al., 2019 [35]	Northern Ireland	Cross-sectional	Participants in the Prospective Epidemiological Study of Myocardial Infarction (PRIME), which is a longitudinal cohort study of cardiovascular disease in Northern Ireland	1380 (male only)	Periodontal parameters: CAL, PD, and number of teeth Pulmonary function measurement: spirometry	A 2-fold increase in mean CAL corresponded to a predicted FEV_1_ value of −3.33% (95% CI = −4.80–1.86), with a *p* < 0.001.
Lee et al., 2020 [37]	USA	Cross-sectional	Participants in the third National Health and Nutrition Examination Survey (NHANES III; 1988–1994),	10,645	Periodontal parameters: PD and LOA Pulmonary function measurement: spirometry	There was a significant inverse correlation between pulmonary function (predicted FEV_1_%, predicted FVC%, and FEV_1_/FVC) and the severity of periodontitis (*p* < 0.001).
Chen et al., 2022 [38]	USA	Cross-sectional	Participants in the National Health and Nutrition Examination Survey (NHANES 2009–2012)	6313	Periodontal parameters: PD and LOA Pulmonary function measurement: spirometry	The ORs for airflow obstruction (FEV_1_/FVC < 0.70) in moderate and severe periodontitis were 1.38 (95% CI = 1.01–1.75) and 1.47 (95% CI = 1.06–2.01).
Lee et al., 2019 [40]	Korea	Cross-sectional	Participants in the sixth Korea National Health and Nutrition Examination Survey (KNHANES; 2014)	4004	Periodontal parameter: CPI Pulmonary function measurement: spirometry	No statistically significant association was found (adjusted OR = 1.140, 95% CI 0.849–1.530) between periodontitis and obstructive pulmonary function impairment (FEV_1_/FVC < 0.7).
Henke et al., 2016 [41]		Cross-sectional	Patients consulting a general dental practice	206	Periodontal parameter: periodontal screening index Pulmonary function measurement: spirometry	After adjustment for covariates, periodontitis was not significantly associated with spirometric measurements (FEV_1_, FVC, FEV_1_/FVC, and peak expiratory flow).
Hämäläinen et al., 2004 [39]	Finland	Cross-sectional and prospective cohort	Participants in the population-based prospective epidemiological cohort study on health and functional capacity (the Evergreen project)	203	Periodontal parameters: BOP, calculus and deepened periodontal pockets Pulmonary function measurement: spirometry (standing position)	At baseline, edentulous male participants had the lowest FEV_1_. After five years of follow-up, the decline in FEV_1_ was greatest in participants with periodontitis or edentulism (−9.4%), whereas no decline was observed in those with healthy periodontal tissue (+1.0%, *p* < 0.006).
Si et al., 2012 [30]	China	Case-control	Patients being treated in the respiratory and dental departments of eight hospitals in Beijing	1019 (case, *n* = 581; control, *n* = 438)	Periodontal parameters: PD, LOA, BI, PI, and ABL Pulmonary function measurements: spirometry and 6-minute walk test	PD, LOA, PI, ABL, and the number of teeth were significantly associated with all stages of COPD (all *p* < 0.001). Patients with higher BODE scores had significantly higher LOA (*p* < 0.001), BI (*p* = 0.027), PI (*p* < 0.001), ABL (*p* < 0.001), and fewer number of teeth (*p* < 0.001).
Peter et al., 2013 [32]	India	Case-control	The case group included well-functioning and ambulatory patients having COPD. The control group consisted of systemically healthy individuals enrolled from the outpatient clinic of the periodontics department.	501 (case, *n* = 102; control, *n* = 399)	Periodontal parameters: OHI, PI, GI, PD, and CAL Pulmonary function measurement: spirometry	Patients with COPD had significantly higher CAL, PD, and OHI than healthy individuals (*p* < 0.0001). A significant negative relationship was found between three periodontal indices (CAL, PD, and GI) and FEV_1_ (*p* < 0.0001).
Tan et al., 2019 [34]	China	Case-control	Participants in a hospital-based study of consecutive cases of COPD at 4 hospitals in Shenyang	160 (case, *n* = 80; control, *n* = 80)	Periodontal parameters: OHI-S, SBI, PD, and CAL Pulmonary function measurement: spirometry	Significant negative correlations of OHI-S (*r* = −0.748, *p* < 0.01) and CAL (*r* = −0.571, *p* < 0.01) with FEV_1_ were observed in the COPD group. Significant negative correlations of OHI-S (*r* = −0.422, *p* < 0.01), SBI (*r* = −0.239, *p* = 0.03), and CAL (*r* = −0.465, *p* < 0.01) with FEV_1_ were also noted in the control group.
Javaheri et al., 2020 [36]	Iran	Case-control	Participants selected from patients with stable COPD with a history of smoking (case) and no pulmonary symptoms with normal spirometry (control) in the same hospital, matched for age and number of teeth	71 (male only: case, *n* = 35; control, *n* = 36)	Periodontal parameters: PD, BOP, and LOA Pulmonary function measurement: spirometry	The PD, BOP, and LOA were negatively correlated with the predicted FEV_1_% (*r* = −0.53, *p* = 0.001), (*r* = −0.62, *p* = 0.001), and (*r* = −0.72, *p* = 0.001) as well as FEV_1_/FVC ratio (*r* = −0.45, *p* = 0.007), (*r* = −0.47, *p* = 0.004) and (*r* = −0.61, *p* = 0.001), respectively.
Hayes et al., 1998 [33]	USA	Nested case-control	Participants in the Veterans Affairs dental longitudinal study and normative aging study	1118 (male only)	Periodontal parameter: radiographic ABL Pulmonary function measurement: spirometry (those whose FEV_1_ was less than 65% of the predicted value were defined as having COPD)	Whole-mouth bone loss was a risk factor for developing COPD (RR = 1.6, 95%CI = 1.2–2.0).
Takeuchi et al., 2019 [42]	Japan	Prospective cohort	Community-dwelling adults without COPD (participants from the Hisayama study)	900	Periodontal parameters: PD, CAL, and the number of teeth Pulmonary function measurement: spirometry	The risk of developing COPD was positively correlated with the severity of periodontitis (*p* for trend = 0.043) after adjusting for smoking intensity and other covariates. The adjusted RR for developing COPD was significantly higher in patients with severe periodontitis than in those with mild periodontitis (RR = 3.51, 95% CI = 1.15–10.74).

ABL, alveolar bone loss; BI, bleeding index; BOP, bleeding on probing; CAL, clinical attachment loss; CI, confidence interval; COPD, chronic obstructive pulmonary disease; CPI, community periodontal index; FEV_1_, forced expiratory volume in one second; FVC, forced vital capacity; GI, gingival index; IRR, incidence rate ratio; LOA, loss of attachment; OHI-S, Oral Hygiene Index—Simplified; OR, odds ratio; PD, probing depth; PI, plaque index; RR, relative risk; SBI, sulcus bleeding index; SpO_2_, percutaneous oxygen saturation.

A prospective, 5-year population-based cohort study in Japan investigated the association between periodontal disease and COPD development in 900 community-dwelling adults without COPD [42]. Twenty-two participants (2.4%) developed COPD during the follow-up period. The risk of developing COPD was significantly increased in participants with severe periodontitis compared with those with no/mild periodontitis (RR = 3.55, 95% CI = 1.18–10.67). The relationship between severe periodontitis and the risk of COPD remained significant (RR = 3.51, 95% CI = 1.15–10.74) after adjusting for potential confounders, including smoking intensity, sex, age, occupation, diabetes mellitus, body mass index, physical activity, alcohol intake, and the number of intact teeth. As the periodontal disease worsened, the risk of developing COPD increased. For example, the risk of developing COPD was 3.2, 4.3, and 13.8 times higher in nonsmokers, moderate smokers, and heavy smokers with severe periodontitis, respectively, compared with nonsmokers with nonsevere periodontitis (controls).

Overall, periodontal disease appears to promote a decline in respiratory function in patients with COPD and other populations; however, the number of prospective studies is limited. Changes in confounding factors during the follow-up period should also be considered in studies with long-term follow-up.

### 2.3. The Influence of Periodontal Disease on QOL Deterioration

The impact of poor oral hygiene on the quality of life of COPD patients is controversial. A cross-sectional study by Zhou et al. examined the relationship between periodontal status and QOL in patients with COPD, as per the St George’s Respiratory Questionnaire (SGRQ) score, a widely used respiratory disease QOL questionnaire. Poor periodontal health (as expressed by the number of missing teeth) and PI were associated with lower QOL. When the researchers adjusted for age, sex, body mass index, and smoking status, missing teeth were significantly associated with symptom and activity scores, and PI was significantly associated with symptom scores [43].

In a cross-sectional study, Baldomero et al. evaluated the correlation between the five-item version of the Oral Health Impact Profile (OHIP)—the most widely used oral health-related quality of life measure—to assess the impact of oral health diseases and dental interventions, establishing SGRQ scores among COPD exacerbators and non-exacerbators. They observed that worse OHIP-5 scores were strongly associated with worse SGRQ scores [44].

A prospective observation by Gaeckle et al. showed that patients with COPD exhibited fewer teeth, a tendency toward a higher PI, and poorer oral health-related quality of life compared to healthy controls. However, PI did not show a significant correlation with daily respiratory symptoms. Interestingly, after controlling for current smoking status, the number of teeth was significantly and positively correlated with the percentage of days with coughing and wheezing on the linear regression analysis. The authors speculated that in patients with poor dental health, diseased teeth may serve as a reservoir for pathogenic microorganisms and inflammatory mediators. Once aspirated into the lower airways, symptoms will likely worsen [45].

Poor periodontal status may exacerbate the QOL of patients with COPD, but one randomized controlled trial did not show any significant improvement in QOL with periodontal treatment. Further studies with a larger number of patients are needed (Table 2).

### 2.4. Periodontal Disease and COPD Exacerbations

Although COPD is primarily a chronic process, many patients experience “exacerbations” wherein their respiratory symptoms worsen rapidly, necessitating therapeutic modifications [47]. COPD exacerbations substantially impact patients’ general health, pulmonary function, and survival [48]. Therefore, preventing exacerbations may improve patients’ QOL and prognoses. COPD exacerbations are mainly triggered by bacterial and viral infections and environmental factors such as air pollution [49]. However, the association between poor oral hygiene and the exacerbation of COPD has also been discussed (Table 3).

Shen et al. carried out a 1:1 propensity-matched cohort study using Taiwan’s national health insurance claims data and showed that adverse respiratory event rates including exacerbations, acute respiratory failure, emergency room visits, hospitalization, and intensive care unit (ICU) admissions were lower in patients with COPD who received periodontal treatment (treatment group) than in patients with COPD but who did not have periodontal disease (control group). The all-cause mortality rate was also favorable in the treatment group [50]. However, as the authors acknowledge, an appropriate control group should be COPD patients comorbid with periodontal disease who are not undergoing periodontal treatment.

Liu et al. investigated the associations of oral hygiene and periodontal health with the exacerbation of COPD in a cross-sectional study. After stratification by smoking, the following were significantly associated with COPD exacerbation frequency: fewer remaining teeth (OR, 2.05) and less frequent tooth brushing (OR, 4.90) in ex-smokers, and higher PI score (OR, 3.43) in never-smokers [51]. A case-control study by Baldomero et al. compared oral hygiene in COPD exacerbators (defined as having at least one exacerbation within the previous year) and non-exacerbators (having no exacerbations within the previous two years). The likelihood of experiencing a severe COPD exacerbation (requiring an emergency room visit and/or hospitalization) was higher for those with more severe periodontitis compared with those who had better dental exam measurements, although the difference did not reach statistical significance [44].

Using data from individuals participating in the Dental Atherosclerosis Risk in Communities Study in the United States, Barros et al. found a statistically significant association between oral health status and COPD-related events (hospitalization for COPD exacerbation or COPD-related death) after adjusting for hypertension, smoking, and diabetes (*p* < 0.0001); the event rates showed a gradient associated with worse oral health status, from 10.5% in those with teeth and healthy periodontium to 23.8% in those with severe periodontal disease, with event rates being highest in edentulous individuals (43.9%) [52]. However, when restricted to only mild or severe periodontal disease, the association was not significant; only edentulism remained a significant risk factor, with a hazard ratio (HR) of 2.28 (95% CI: 1.46–3.56).

The impact of periodontal disease on the frequency of COPD exacerbations remains controversial, with limited reports. Further interventional investigations, such as those described below, are needed.

**Table 3:** Studies evaluating the link between periodontal disease and the risk of COPD exacerbation.

Author (Year)	Location	Study Design	Study Population	*n*	Measured Outcome	Main Findings
Shen et al., 2009 [50]	Taiwan	Retrospective cohort study (1:1 propensity score matching)	The National Health Insurance claims data from the National Health Research Institutes of Taiwan	Patients with COPD receiving periodontal treatment (*n* = 5562) vs. COPD patients without periodontal disease (*n* = 5562) The periodontal treatment included a basic form of subgingival curettage (scaling, root planing) and an invasive form of periodontal flap surgery.	Definition of adverse respiratory event: emergency room visit or hospitalization due to exacerbation of COPD, pneumonia, and acute respiratory failure	Emergency room visits for adverse respiratory events; 3.79 (treatment group) vs. 4.21 (control group) per 100 person-years, with adjusted IRR of 0.86 (95% CI = 0.78–0.94, *p* < 0.01) Hospitalizations for adverse respiratory events; 2.75 (treatment group) vs. 3.65 (control group) per 100 person-years, with an adjusted IRR of 0.74 (95% CI = 0.69–0.80, *p* < 0.001). ICU admission; 0.66 (treatment group) vs. 0.75 (control group) per 100 person-years with an adjusted IRR of 0.84 (95% CI = 0.75–0.94, *p* < 0.01). All-cause mortality; 1.81 (treatment group) vs. 2.87 (control group) per 100 person-years, with an adjusted RR of 0.57 (95% CI = 0.52–0.62, *p* < 0.001)
Liu et al., 2012 [51]	China	Cross-sectional	Ambulatory patients with COPD, treated at eight hospitals in Beijing (frequent exacerbator vs. infrequent exacerbator)	392 (frequent exacerbator, *n* = 183; infrequent exacerbator, *n* = 209)	Periodontal parameters: PD, CAL, BOP and PI Definition of COPD exacerbation: the presence of two or more of the following symptoms and a change in medication; increased dyspnoea, cough, sputum volume, or sputum purulence compared with their baseline status Definition of frequent exacerbator: those who experienced two or more exacerbations in the last 12 months	Fewer remaining teeth (*p* = 0.02), higher PI scores (*p* = 0.02), and less frequent tooth brushing (*p* = 0.008) were statistically significantly associated with COPD exacerbations. When stratified by smoking, higher PI scores (OR = 3.43, 95% CI = 1.19–9.94) were significantly associated with COPD exacerbations in never-smokers.
Baldomero et al., 2019 [44]	USA	Case-control (exacerbators vs. non-exacerbators)	Individuals from the Minneapolis Veterans Affairs health care system	136 (patients with COPD: exacerbator, *n* = 70; non-exacerbator, *n* = 66)	Periodontal parameters: OHIP-5; PD, CAL, BOP, GI, PI, and caries risk assessment (subset of patients) Definition of exacerbator: at least one COPD exacerbation in the previous 12 months Definition of COPD exacerbation: taking antibiotics and/or oral corticosteroids for respiratory symptoms or hospitalization for respiratory symptoms or emergency room visit for respiratory illness	Unadjusted odds ratios for severe exacerbations to mild exacerbations tended to be higher for those with worse measures of periodontitis severity, PD, CAL, BOP, PI, and GI, and caries risk assessment, but the difference was not statistically significant. Due to the small sample size, adjustment for covariates was not performed.
Barros et al., 2013 [52]	USA	Prospective cohort	Participants in the Dental Atherosclerosis Risk in Communities study	1635 (patients with COPD: individuals with COPD-related events at 5-year follow-up, *n* = 399; individuals without events at 5-year follow-up, *n* = 1236)	Periodontal parameters: PD, CAL, and the number of teeth Definition of COPD-related event: hospitalization due to COPD exacerbation or COPD-related death	There was a statistically significant association between oral health status and COPD-related events (*p* < 0.0001). The event rates showed a gradient associated with worse oral health status, ranging from 10.5% in those with teeth and healthy periodontium to 23.8% in those with severe periodontal disease, with the highest event rate in the edentulous (43.9%).

BOP, bleeding on probing; CAL, clinical attachment loss; CI, confidence interval; COPD, chronic obstructive pulmonary disease; GI, gingival index; ICU, intensive care unit; IRR, incidence rate ratio; OHIP, Oral Health Impact Profile; OR, odds ratio; PD, probing depth; PI, plaque index; RR, relative risk.

### 2.5. Impact of Therapeutic Interventions for Periodontal Disease on the Health Outcome of COPD

Several studies have examined whether periodontal interventions can improve outcomes in COPD, but the results are controversial (Table 4).

In a prospective, controlled group trial, Kucukcoskun et al. compared the frequency of exacerbations in 40 patients with COPD and at least one infective exacerbation within the previous year. Participants received three periodontal treatments at 1-week intervals (experimental group) or served as no-treatment controls. The initial periodontal treatment included oral hygiene instructions, full-mouth scaling, and root planing using handheld instruments and ultrasonic devices. The initial periodontal treatment group showed a significant decrease in exacerbation frequency during the 1-year follow-up period (median: 3 ± 1.83 before the intervention, 1.95 ± 1.46 after intervention), while no significant decrease was observed in the control group [53].

Sharma et al. prospectively examined the influence of nonsurgical periodontal therapy on the periodontal clinical parameters and pulmonary function in patients with COPD and chronic periodontitis, compared with systemically healthy controls who also demonstrated chronic periodontitis. After 12 months of treatment, the periodontal parameters of mean PI, GI, PD, CAL, and BOP were significantly improved in both groups. Moreover, the FEV_1_/FVC values improved significantly in patients with COPD plus chronic periodontitis (15.85% improvement from baseline); in contrast, the control group showed no significant changes [54].

Das et al. conducted a randomized controlled trial comparing patients with COPD (*n* = 18) treated with full-mouth scaling and root planing (SRP) using hand instruments to a control group (no treatment, *n* = 17). The intervention group showed significant improvement in SGRQ activity scores after one year of treatment (mean ± standard deviation, with 53.68 ± 16.37 before treatment vs. 38.20 ± 13.18 after treatment, *p* = 0.005), whereas the control group showed no change [55].

Zhou et al. conducted a 2-year pilot randomized controlled trial of 60 patients with COPD and chronic periodontitis. The participants were divided into three groups. One group underwent SRP. Another underwent supragingival scaling treatment. The third group only received oral hygiene instruction and no periodontal treatments. Patients in the two treatment groups showed a significant improvement in periodontal index measurements compared with the control group at all follow-up time points. Mean FEV_1_/FVC and FEV_1_ values were also significantly higher for the two treatment groups. Moreover, the proportion of patients with frequent COPD exacerbations was significantly lower in the two treatment groups than in the control group at 2 years: SRP group, 30.0%; supragingival scaling group, 15.8%; and control group, 66.7% [56]. Though preliminary, the study’s results suggested that periodontal treatment may mitigate the risk of functional pulmonary deterioration over time in patients with COPD and periodontitis.

**Table 4:** Effects of periodontal interventions on COPD outcomes.

Author (Year)	Location	Study Design	Study Population	*n*	Periodontal Intervention	Measured Outcome of COPD	Main Findings
Madalli et al., 2016 [57]	India	Prospective cohort	Patients diagnosed with COPD and chronic periodontitis	30	Supragingival scaling and oral hygiene instructions to all patients	Spirometric data (FEV_1_ and FVC)	No statistically significant improvement in FEV_1_/FVC values was observed before (mean ± SD, 48.08 ± 12.01) and after treatment (51.25 ± 12.29, *p* > 0.05).
Kucukcoskun et al., 2013 [53]	Turkey	Prospective case–control	Patients with COPD attending the outpatient clinics of the three chest clinics with a history of at least one infectious exacerbation in the past year and with moderate to severe chronic periodontitis Treatment group: patients who were able to visit the authors’ department regularly for treatment and subsequent follow-up Control group: patients who came from hospitals distant from the authors’ periodontology department and had transportation problems	40	Oral hygiene instructions, full-mouth scaling, and root planing using hand instruments and ultrasonic devices under local anaesthesia, *n* = 20; no periodontal treatment, *n* = 20	Rate of exacerbation (sustained worsening of baseline respiratory symptoms for ≥2 days that required oral corticosteroids and antibiotics/hospitalization) over the 12 months	Exacerbation frequency was significantly reduced in the treatment group (mean ± SD, 3 ± 1.83 at baseline; 1.95 ± 1.46 after 1-year follow-up; *p* = 0.01), whereas there was no change in the number of exacerbations in the control group. (3.5 ± 4.62 at baseline; 3.25 ± 3.35 after 1-year follow-up; *p* = 0.87).
Sharma et al., 2021 [54]	India	Prospective case–control	Case group: patients with COPD having chronic periodontal disease and a history of exacerbation within the last month Control group: systemically healthy outpatients with periodontitis	75	Non-surgical periodontal therapy: oral hygiene instructions and professional full mouth SRP using an Ultrasonic scaler and periodontal hand instruments without local anaesthesia, *n* = 37; no periodontal treatment, *n* = 38	Spirometric data (FEV_1_ and FVC)	The case group showed a statistically significant improvement in mean FEV_1_/FVC values at 12 months (mean ± SD, 63.39 ± 11.65 vs. 73.43 ± 10.51; *p* < 0.001). In contrast, no significant changes were observed in the control group (mean ± SD, 82.49 ± 5.29 vs. 81.49 ± 3.82; *p* = 0.485).
Das et al., 2017 [55]	India	Randomized controlled trial	Patients with COPD	35	Full-mouth scaling and root planing using hand instruments, and oral hygiene instructions, *n* = 18; no periodontal treatment, *n* = 17	SGRQ	The intervention group showed a significant improvement in the activity score after 1 year of treatment (mean ± SD, 53.68 ± 16.37 before treatment vs. 38.20 ± 13.18 after treatment; *p* = 0.005), whereas there was no change in the control group.
Zhou et al., 2014 [56]	China	Randomized controlled trial	Symptomatic patients with COPD attending a hospital in Beijing	60	SRP treatment, *n* = 20; supragingival scaling treatment, *n* = 20; no periodontal treatment, *n* = 20	Pulmonary function (FEV_1_ % predicted, FEV_1_/ FVC) and the frequencies of COPD exacerbation	The mean FEV_1_ values of the two treatment groups were significantly higher (*p* = 0.03) than the control group at 1-year follow-up, although the difference did not reach statistical significance at a 2-year follow-up visit (*p* = 0.06). The FEV_1_/FVC means of the two treatment groups were higher than the control group at 1 year (*p* = 0.04) and at the 2-year follow-up (*p* = 0.02), respectively. At the 2-year follow-up visit, the treatment group had a lower rate of frequent exacerbations (SRP, 30%; supragingival scaling treatment, 15.8%) than the control group (66.7%), which was statistically significant between groups (*p* = 0.004). Adjusted ORs for frequent exacerbation were 0.29 (95% CI: 0.10–0.84) for the SRP group and 0.04 (95% CI: 0.003–0.64) for the supragingival scaling group.
Agado et al., 2012 [46]	USA	Randomized controlled trial	Patients diagnosed with COPD and chronic periodontitis	30	Magnetostrictive ultrasonic instrument, *n* = 10; hand instrument, *n* = 10; control, *n* = 10	SGRQ-A and Illness Questionnaire (developed by the principal investigator)	SGRQ-A (symptom, *p* = 0.124; activity, *p* = 0.702; impact, *p* = 0.926) and illness questionnaire scores did not demonstrate significant differences in QOL or illness after periodontal debridement between groups.
Sundh et al., 2021 [58]	Sweden	Randomized controlled trial	Patients with COPD recruited at hospitals and primary healthcare centers	101	Advanced dental cleaning (modification of the full-mouth disinfection protocol), *n* = 45; control (dental examination and supra-gingival cleaning using toothpaste, corresponding to tooth brushing), *n* = 56	Exacerbation frequency, pulmonary function (FEV_1_ % predicted), and CAT score	The frequency of annual exacerbations was significantly reduced (*p* = 0.039) in patients who underwent repeated advanced dental cleanings (median, −1.0; IQR, −3.5–0.0), although no significant differences were found in the CAT score and FEV_1_ % predicted.

CAT, COPD Assessment Test; CI, confidence interval; COPD, chronic obstructive pulmonary disease; FEV_1_, forced expiratory volume in one second; FVC, forced vital capacity; IQR, interquartile range; OR, odds ratio; QOL, quality of life; SGRQ, St. George’s Respiratory Questionnaire; SRP, scaling and root planing; SD = standard deviation.

On the other hand, other studies produced negative results. Madalli et al. examined the effect of supragingival scaling and oral hygiene instructions on patients with COPD complicated by periodontal disease (*n* = 30) in a prospective cohort study but found no significant improvement in pulmonary function [57]. Sundh et al. conducted a randomized trial comparing an intervention group whose members underwent advanced tooth cleaning (*n* = 45) to a control group (*n* = 56). Patients who received repeated advanced dental cleanings or examinations showed a significant reduction in exacerbation frequency controls; however, the difference did not reach significance in the intent-to-treat analysis [58]. One randomized controlled pilot study compared three groups (*n* = 10 each) of patients with COPD and chronic periodontitis. One-third received magnetostrictive ultrasonic instrumentation, one-third received handheld instrumentation, and one-third served as a nontreated control group. There were no significant between-group differences in QOL and illness scores (as measured by the SGRQ and the principal investigator’s original illness questionnaire) [46]. The authors concluded that periodontal debridement did not affect QOL in patients with COPD and chronic periodontitis.

Examining the potential for improving COPD outcomes through therapeutic interventions for periodontal disease is important in terms of providing additional therapeutic options for the disease. However, most of the aforementioned studies are limited by small sample sizes and variability in the treatment methods and durations. When evaluating exacerbations as an outcome, other factors contributing to exacerbations (e.g., peripheral blood eosinophils or a history of previous exacerbations), medications used (inhaled corticosteroid use or not), and medication adherence must also be considered. Well-powered randomized trials that overcome these issues are needed.

## 3. Biological Mechanisms Underlying This Relationship

### 3.1. Common Genetic Predisposition

In a common susceptibility model proposed by Seymour et al., it is hypothesized that some individuals harbor a predisposition to both periodontal and systemic diseases [59]. Yu and coworkers investigated the association between single-nucleotide polymorphisms (SNPs) in toll-like receptor (TLR)-4 in Han Chinese patients with chronic periodontitis and COPD. Genotyping of six candidate SNPs in TLR4 was performed in 339 patients with chronic periodontitis only and in 373 patients with chronic periodontitis and COPD. The results showed that patients with chronic periodontitis who harbored the AG polymorphism of TLR4 rs1927907 were significantly more likely to develop COPD complications than patients with the GG genotype (OR, 1.94) after adjusting for age, sex, smoking, and oral hygiene habits [60]. Liu et al. evaluated the pathophysiological interrelation between chronic periodontitis and COPD using the Gene Expression Omnibus database and found that EPB41L4A-AS1, INSR, and R3HDM1 could act as cross-talk genes between the two diseases [61]. These findings need to be further evaluated in experimental and clinical studies.

### 3.2. Other Shared Risk Factors

Both periodontal disease and COPD share common risk factors, including aging and cigarette smoking [16]. One epidemiological study found that the prevalence of chronic periodontal disease is highest among older adults (82%), followed by adults (73%) and adolescents (59%) [62]. In addition, periodontal disease severity increases with advancing age [63]. Similarly, COPD is a condition that is more likely to develop with aging [3]. Cigarette smoking, the main risk factor for COPD, also increases the risk of periodontal disease by 5 to 20 times [64]. Low socioeconomic status is another common risk factor for COPD and periodontitis [65,66,67,68]. The association between these diseases and socioeconomic status may reflect poor oral health and other factors such as smoking, poor nutrition, inadequate self-care behavior, and air pollution [16].

In contrast, other factors are being reported that may link the two diseases. A case-control study by Zhou et al. showed that serum levels of 25-hydroxyvitamin D [25(OH)D], the major metabolite of circulating vitamin D, correlated positively with pulmonary function in nonsmokers and correlated negatively with PI in ex-smokers. The periodontal indices were significantly correlated with the serum level of 25(OH)D (PD, CAL, BI, PI, and ABL in the COPD group) when adjusted for age, gender, body mass index, season, and smoking status [69]. Previous studies have observed that vitamin D potentially decreases gingival susceptibility to inflammation due to its anti-inflammatory effects [70,71]. Moreover, vitamin D insufficiency was more common in patients with COPD and correlated with FEV_1_ [72,73]. Therefore, low vitamin D levels may be involved in developing COPD and periodontal disease.

There are several possible mechanisms for vitamin D insufficiency inducing COPD. Some epidemiological studies have documented an association between vitamin D insufficiency and an elevated incidence of respiratory tract infections [74] and airway bacterial colonization [75]. Vitamin D functions as an important activator of innate immunity through the generation of antimicrobial peptides (cathelicidin and defensins) upon the stimulation of TLRs by the lipopeptides of pathogens [76]. Airway infection due to vitamin D insufficiency may contribute to the development and exacerbation of COPD. Genetic conditions for vitamin D-binding proteins may also influence the body’s susceptibility to COPD [77]. Vitamin D is involved in the homeostasis of the extracellular matrix in tissues other than bone, such as in the lungs, through the regulation of transforming growth factor-b and matrix metalloproteinase (MMP) [78]. This effect may participate in lung tissue remodeling [69]. Reportedly, vitamin D, 25(OH)D, and 1,25(OH)2D significantly reduced the production of prostaglandin E2 (PGE2), a known inhibitor of fibroblast repair functions, in human lung fibroblasts [79]. Elevated PGE2 concentrations have been reported in the lower airways of patients with COPD. Fibroblasts are the main source of PGE2 in the lungs, and it is also known that fibroblasts from patients with COPD overproduce PGE2 [80]. This excessive production is partly responsible for the reduced repair response seen in COPD fibroblasts. Therefore, vitamin D insufficiency, leading to the excessive production of PGE2, may be involved in the pathogenesis of COPD [79]. In addition, numerous studies have suggested that vitamin D insufficiency may play a role in the pathogenesis of periodontal disease [81]. Han et al. examined the effects of vitamin D supplementation in a rat model of COPD when comorbid with periodontitis. The results showed that 25-hydroxyvitamin D3 treatment significantly alleviated inflammation, reduced ABL, and slightly improved lung function, with decreased serum levels of the receptor activator of the nuclear factor κ-B ligand (RANKL), tumor necrosis factor-alpha, and interleukin (IL)-1, along with increased IL-10 [82].

### 3.3. The Role of Microorganisms

The aspiration of oral bacteria can exacerbate lower respiratory tract disease [83]. Pathogen-derived proinflammatory cytokines have a key role during the exacerbation of COPD. Periodontal pathogens such as *P*. *gingivalis* and *Fusobacterium nucleatum*, even when heat-inactivated, produce large amounts of inflammatory cytokines, such as IL-6 and IL-8, from the human epithelial cell lines derived from the bronchi, alveoli, and pharynx [84,85]. The tracheal inoculation of periodontal bacteria increases the production of IL-6 and KC (an isoform of mouse IL-8) in the lungs and bronchi and also increases the levels of these cytokines in the serum in mice [85]. Tian et al. showed an enhanced accumulation of inflammatory cytokines and neutrophils in the lung tissue and blood in a mouse model of ligature plus *P*. *gingivalis*-induced periodontitis. They speculated that gingipain, a protease produced by *P*. *gingivalis*, was an essential component [86]. Periodontal pathogens may be involved in worsening inflammation, airflow limitation, and the destruction of lung structures. *P*. *gingivalis* and *F*. *nucleatum* induce the gene expression of MUC5AC, a mucin core protein, in primary human bronchial epithelial cells, potentially causing airway lumen narrowing [87,88]. Intratracheal inoculations of *F*. *nucleatum* to elastase-induced emphysematous mice resulted in a progression of the inflammatory response and increased mean interalveolar distance, indicating the breakdown of alveolar walls, leading to emphysema and the recruitment of mucin, all of which contribute to the pathogenesis of COPD. The authors of this study also demonstrated the increased expression of MMP-12, which is involved in the destruction of alveolar walls [89]. The same research group has also shown that the administration of *F*. *nucleatum* increases the expression of MMP-9, which is involved in the inflammatory response and in the degradation of components of the lung extracellular matrix such as elastin and collagen [90], in an A549 cell culture and in mouse lung and bronchoalveolar lavage fluid [91]. Yoshida et al. showed that the extracellular vesicles derived from *P*. *gingivalis*-infected macrophages induced pulmonary inflammation and alveoli destruction in mice [92].

Conversely, Rigauts et al. reported that *Rothia mucilaginosa*, a common microorganism found in the oral cavity, exhibits inhibitory activity against the inflammatory reactions induced by pathogens and lipopolysaccharides, both in vitro (in a three-dimensional cell culture model) and in vivo (in a mouse model) [93]. The pathogen is also known to have been detected in COPD patients’ lungs [94]. However, the involvement of this pathogen in the pathogenesis of COPD remains to be elucidated.

In a prospective observational study, Tan et al. explored the homology of bacteria in dental plaques and tracheal aspirate samples from 53 patients admitted to the ICU with COPD exacerbations, using 16S ribosomal DNA (rDNA)-based metagenomic analysis. Three pathogens that are involved in COPD exacerbations, namely, *Pseudomonas aeruginosa*, *Klebsiella pneumoniae*, and *Streptococcus pneumoniae*, were detected in both the tracheal aspirate and the corresponding dental plaque samples, with a high degree of homology (97–100% similarity in the 16S rDNA sequence). Based on these results, the authors proposed that oral plaque may be a reservoir of pathogenic respiratory bacteria [95]. Wu and coworkers examined oral microbiota using 16S rRNA gene metagenomic sequencing in the following four groups: 30 patients with periodontitis and COPD (C+P+ group), 25 patients with COPD without periodontitis (C+P− group), 25 patients with periodontitis without COPD (C−P+ group), and 25 healthy individuals (C−P− group). The results showed that the periodontitis-associated genera of *Dysgonomonas*, *Desulfobulbus*, *Catonella*, and four species (*Porphyromonas endodontalis*, *Dysgonomonas wimpennyi*, *Catonella morbi*, and *Prevotella intermedia*) were increased in patients with both COPD and periodontitis. The authors concluded that the abundant presence of these pathogens in periodontal tissues may be related to the development of COPD [96]. In the study by Lin et al., the salivary microbiota of patients with COPD and periodontitis was compared with that of patients with periodontitis only and that of healthy individuals, using 16s rRNA gene sequencing. They reported that the bacterial family of Lachnospiraceae was frequently observed only in patients with COPD and periodontitis. *Veillonella*, *Rothia*, and *Actinomyces* were observed more frequently in patients with COPD and periodontitis than in healthy individuals [97]. Khijmatgar et al. reported that oral Candida was more common in the COPD group than in the controls (21.42% vs. 1.1%). They also observed higher DMFT and significant caries index scores in patients with COPD [98].

However, there are also some conflicting reports. A case-control study by Zhou et al. revealed that although patients with COPD had poorer periodontal health (higher PI and CAL) than the controls, no significant differences were found in the detectability of the nine pathogens in subgingival dental plaque samples [99]. Takahashi et al. examined the association between IgG antibody levels against *P*. *gingivalis*, the most frequently found pathogen in patients with periodontitis, and the frequency of COPD exacerbations. Contrary to their hypothesis, participants with high IgG titers for *P*. *gingivalis* had significantly fewer exacerbations (mean: 0.8 vs. 1.2/year) and a lower proportion of frequent exacerbators (at least twice a year) compared with those participants with normal immunoglobulin (Ig) G titers (14.3% vs. 38.6%). They suggest that the lack of *P*. *gingivalis*-associated antibodies contributes to the frequency of exacerbations [100]. Meanwhile, the limitation of their study was that the antibody measurements were performed only once, and the severity of periodontitis, antibody titers, and the immune status of the host might vary over the study period, as the authors acknowledged.

Although oral microorganisms including *P*. *gingivalis* and *F*. *nucleatum* may facilitate the development of COPD through neutrophils and MMPs, there are conflicting reports in the literature, and further research is needed to clarify the detailed mechanisms. Further studies are also warranted to elucidate whether effective periodontal therapy differs for each microorganism.

### 3.4. Therapeutic Agents

Inhaled drugs such as long-acting muscarinic antagonists, long-acting beta-2 agonists, and inhaled corticosteroids (ICS) are the mainstay treatments for COPD. Theophylline and oral corticosteroids may also be used in adjunctive therapy. The impact of these inhaled and oral medications on oral hygiene has been widely investigated in individuals with asthma [101,102,103,104]. Beta-2 agonists are responsible for a decreased salivary rate, a lower buffering capacity of saliva, and lower oral pH [105,106,107,108]. Treatment with ICS promotes the growth of Candida species and other microorganisms via elevated glucose levels in saliva [109,110,111]. Decreased salivary IgA due to ICS may also contribute to the growth of bacteria [112]. Anticholinergics may also decrease salivary production and can interfere with the clearance of oral bacteria [113]. Theophylline medication can be a risk factor for increased candidal load in patients with COPD [98].

Bozejac et al. investigated the impact of inhalation therapy in an adult population. The results showed that the salivary flow rate was significantly lower in patients on inhaled medications compared with the control group, although this study included patients with asthma and COPD [114]. In a nationwide population-based cohort study in Taiwan, patients with COPD who received ICS (HR, 1.22) and systemic corticosteroid treatment (HR, 1.15) had an increased risk of periodontal disease compared with those patients who did not receive corticosteroid treatment [115].

Most of the previous studies addressing the impact of inhaled medications on oral health have focused on younger people with asthma and dental caries [116]. When focusing on the pathogenesis of COPD, the relationship with periodontal disease is likely to be more complex, due to the effect of additional influences such as aging and smoking; therefore, further research is warranted.

### 3.5. Inflammatory Mediators

Suggested mechanisms for the association between periodontal disease and COPD include the overspill of locally generated inflammatory mediators into the systemic circulation and/or bacteremia of oral or pulmonary origin [16]. Several studies have been conducted on the inflammatory mediators involved in these two diseases.

Öztekin et al. compared the high-sensitivity C-reactive protein (hs-CRP) levels in serum, hs-CRP, IL-1b, and PGE2 in gingival crevicular fluid in patients with COPD and in control individuals. All were significantly higher in patients with COPD, while serum hs-CRP was higher in patients with an increased severity of COPD. Serum hs-CRP levels were positively correlated with gingival crevicular fluid hs-CRP levels and periodontal disease in patients with COPD [117]. Barros et al. observed that the severity of periodontal disease and the high incidence of COPD-related events parallel the elevated inflammatory marker IL-6; they postulate that systemic inflammation may be the origin of these diseases [52]. Interleukin-6 has been reported to be involved in COPD as a disease driver and predictor of exacerbations [118,119,120]. Several reports on IL-11, a member of the cytokine IL-6 family, suggest a link between the two diseases [121]. An alteration in IL-11 gene expression may contribute to a genetic predisposition to COPD [122]. Concentrations of IL-11 were significantly higher in the affected gingiva than in healthy sites in samples taken from the biopsy specimens of patients with periodontitis, indicating a putative function of IL-11 in the gingival tissue during the initial phase of gingival inflammation [123]. Liu et al. summarized the role of T helper 17 cells and the related cytokines, including IL-17, IL-22, IL-1β, IL-6, IL-23, and transforming growth factor-β, in the association between periodontal disease and COPD [116]. These cytokines have been detected in the blood, sputum, gingival crevicular fluid, or lung/periodontal tissues of patients with periodontal disease and COPD and have been shown to contribute, directly or indirectly, to inflammation and tissue damage.

As reported by Usher et al., periodontitis and COPD (especially the emphysema-dominant phenotype) are thought to have a common pathogenesis of inflammation and local connective tissue destruction. Neutrophils are key players in the inflammatory response in both diseases, and their proteases and reactive oxygen species have been shown to spread inflammation and result in connective tissue component destruction. The authors speculated that an imbalance between these degrading proteins and their inhibitors contributes to disease development [124]. Patients with COPD and alpha-1 antitrypsin deficiency (AATD), a disease that causes the development of COPD in young populations due to a deficiency in alpha-1 antitrypsin, had a higher prevalence of periodontitis (COPD 95%, AATD 88%) than the prevalence reported by unselected community residents (data were taken from NHANES and the UK Adult Dental Health Survey in 2009), even after accounting for risk factors. The authors of this study reported that patients with COPD or AATD and stage II–IV periodontitis exhibited declined neutrophil migration accuracy compared to patients with COPD or AATD but who were without (or with stage I) periodontitis, suggesting altered neutrophil function [125]. The authors also speculated that AATD may destroy the periodontal tissue as well as the lungs because the prevalence of periodontitis was high (72%) in AATD patients, despite their having fewer risk factors for periodontitis and good dental habits.

Matrix metalloproteinases-8 and -9 and the tissue inhibitors of matrix metalloproteinase (TIMP)-1 have been implicated in the pathogenesis of COPD and decreased pulmonary function in previous studies [126,127,128]. In addition, MMP-8 and -9 have also been reported as contributing factors in the pathogenesis of periodontal disease [129,130,131,132]. Ji et al. evaluated the inflammatory biomarkers that play an important role in COPD in different airway compartments. They noted a negative correlation between pulmonary function and salivary IL-8 and MMP-9 in smokers with COPD. In addition, salivary IL-8 and MMP-9 were positively correlated with periodontal disease, as assessed by gingival bleeding in nonsmokers [133]. Although these findings raise the possibility that MMP-9 and IL-8 may be involved in both periodontal disease and COPD, the results differ between smokers and nonsmokers, and further investigations are needed.

Despite these results, some reports have raised questions about the influence of these factors. A case-control study in Turkey showed that the amounts of MMP-8 in serum, measured using an immunofluorescence assay, were significantly higher in the mild COPD group than in the control group; however, the amounts of MMP-8 and TIMP-1 in serum, measured using an enzyme-linked immunosorbent assay, were similar in both groups. The values of these parameters in the saliva did not differ between the two groups. There were also no significant between-group differences in the periodontal clinical parameters, including CAL, PI, and BOP [134]. Although Winning et al. showed that chronic periodontitis was associated with decreased pulmonary function, their mediation analysis showed that the mediating effect of hs-CRP was significant, but minor, in mediating the relationship between mean CAL and percentage predicted FEV_1_, with full adjustment for potential confounders. The CRP inflammatory pathway represented only 9% of the total impact of the mean CAL on the percentage predicted FEV_1_ [35]. According to Rosa et al., the induction of periodontitis, per se, does not alter any of the inflammatory parameters, including IL-6 and IL-17, except for an increase in tumor necrosis factor-alpha in the bronchoalveolar lavage fluid in the COPD + periodontitis group compared to the COPD-only group in mice [135].

These observations suggest a need for further research on the systemic inflammatory mediators linking periodontal disease and COPD, although interleukins, neutrophils, and MMPs may be involved in the pathogenesis of both diseases.

### 3.6. Sarcopenia, Masticatory Function and Brushing Behavior

Sarcopenia refers to age-related reductions in muscle mass, muscle strength, and physical function. Sarcopenia is highly prevalent in patients with COPD [136]. Sarcopenia can also lead to the worsening of dyspnea [137]. In this regard, Terashima et al. examined chewing ability in patients with COPD and controls using color-changing chewing gum [138]. Chewing ability was evaluated on a 5-point scale, based on the change in the gum’s color after 1 min. The mean color score of the chewed gum was lower in the COPD group than in the control group. Reduced muscle mass, having fewer than 20 remaining teeth, and COPD were identified as risk factors for impaired chewing ability. Moreover, they also monitored the patients for a decrease in peripheral oxygen saturation (SpO_2_) during gum chewing. The COPD group showed a significantly greater mean SpO_2_ decrease (0.78% ± 1.46%) during 1 min of gum chewing than the control group (0.32% ± 0.72%). There was a significant decrease in the mean SpO_2_ during gum chewing (95.1% ± 2.4%) compared to the level before gum chewing (95.9% ± 1.7%); the decrease in SpO_2_ was greater in patients with COPD with fewer remaining teeth. Patients with COPD whose SpO_2_ decreased by 4% or more during the 6-minute walk test showed greater desaturation when chewing gum. Individuals with low chewing ability consumed a lesser variety of foods and less frequently consumed beans, vegetables, seaweed, and nuts than those with high chewing ability [139], which may lead to malnutrition. The authors hypothesized that patients with sarcopenia and COPD demonstrated poor oral hygiene and impaired nutritional intake. Their case-control study found that patients with COPD had fewer teeth, more BOP and PD, and lower serum albumin concentration than nonsmokers or smokers without COPD [140]. In addition, poor periodontal hygiene is associated with hypoalbuminemia, suggesting that a reduced dietary intake due to fewer teeth may affect the nutritional status of patients with COPD.

If patients with COPD become undernourished due to the above mechanisms, they may develop sarcopenia [141], further affecting their tooth-brushing behavior. Using the Korean NHANES data from 2008 to 2011, Han et al. investigated the oral health status of individuals with and without sarcopenia. They found that individuals with sarcopenia brushed their teeth less frequently. The prevalence of periodontitis was significantly higher among participants with sarcopenia (30.3% of males and 45.9% of females) than among those without sarcopenia (18.3% of males and 17.4% of females). The count of natural teeth was significantly fewer in participants with sarcopenia [142]. Chung et al. investigated oral hygiene in patients with COPD using the Korean NHANES database. Patients with COPD demonstrate less frequent tooth brushing and less frequent use of dental floss, interdental brushes, mouthwash, and electric toothbrushes, compared with individuals without COPD [143]. Infrequent brushing was also observed in a study of patients with COPD in India [144]. Wang et al. also found less frequent tooth brushing, less frequent use of the horizontal tooth-brushing maneuver, and poor oral health knowledge associated with COPD in the Chinese population [145].

Taken together, a vicious cycle can be assumed in which sarcopenia exacerbates dyspnea in patients with COPD, leading to inadequate brushing behavior and poor oral hygiene, further deteriorating COPD through microbial growth and the spillover of inflammation, as well as malnutrition resulting from poor masticatory function.

## 4. Implications for Future Research

Although numerous studies suggest a link between periodontal disease and COPD, several issues remain to be solved. Most reports examining the association between the two diseases have had a cross-sectional or case-control design, and prospective studies are limited. Cross-sectional and case-control studies may be less precise and more susceptible to recall bias than evidence from cohort studies [22]. Several prospective interventional studies have been reported, but the majority of them are based on small cohorts and the number may be insufficient to reach satisfactory conclusions. In some studies, the diagnostic criteria for COPD differ from those currently used. It is also important to interpret the results of studies to ensure that common risk factors for periodontal disease and COPD, such as smoking and socioeconomic status, are appropriately controlled and that the effects of medications and dietary or brushing habits are adequately handled. There are many studies on the relationship between inhaled medications and oral health in children and adolescents, but few studies concern the adult population [114]. Common genetic predisposition has not been widely studied and this should be remedied in the future. When evaluating COPD exacerbations as an outcome, other factors related to exacerbations, such as blood eosinophil count [146], a history of prior exacerbations [147], symptom intensity (i.e., the COPD Assessment Test score), and severe airflow limitation [49,148] should be properly controlled.

Some studies have analyzed smoking-related COPD and never-smoker COPD (such as from biomass burning) together, but their phenotypes may differ. Approximately 30% of individuals with COPD have never smoked [149]. Previous studies have suggested that biomass smoke exposure results in less emphysema formation in the lungs than tobacco smoke exposure but results in more air trapping. This leads to an airway-dominant COPD phenotype [150,151]. Furthermore, there are significant differences in the nasal and oral microbiota between tobacco smoke-associated COPD and biomass smoke-associated COPD [152].

Large randomized controlled trials are needed to determine whether the treatment of periodontal disease reduces COPD exacerbations and death. It is also important to motivate patients to seek oral disease treatment. Devlin drew attention to a major reason why patients are less likely to participate in dental practice appointments: a lack of transportation. Patients with severe COPD experience impaired mobility, breathing difficulties, and frequent hospitalizations. These factors may prevent them from keeping regular dental appointments [153]. Indeed, the most commonly reported reason for patients with COPD not attending pulmonary rehabilitation was transport problems [154]. Considering this finding, telemedicine/teledentistry services [155] offer promising support tools for patients with COPD; however, these tools require further validation. It is also intriguing to investigate whether the treatment of periodontal disease reduces the incidence of extrapulmonary comorbidities of COPD (cardiovascular disease, osteoporosis, gastroesophageal reflux disease, etc.).

Patient education is another important task. Several studies have shown the usefulness of periodontal self-assessment [156,157]. Patients require training to perform such assessments during periodontal disease screenings and should be encouraged to self-manage where possible, particularly in resource-limited situations.

## 5. Conclusions

**Figure 1:**
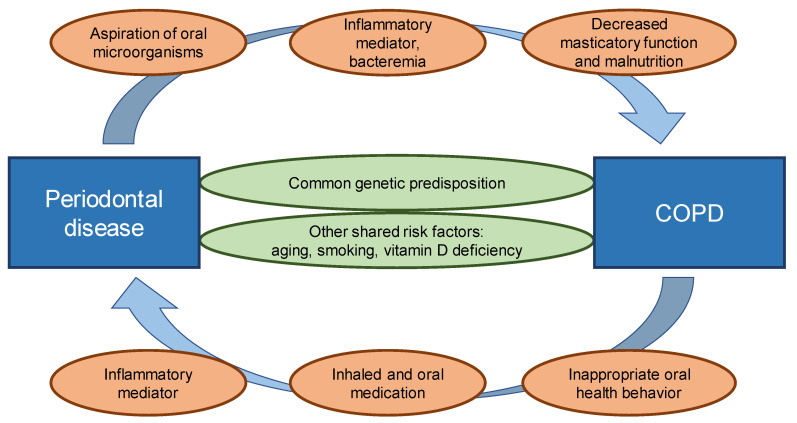
A schematic diagram showing the association between periodontal disease and COPD.

This review describes the relationship between periodontal disease and COPD. The relationship between periodontal health and COPD risk is biologically plausible, and the association may be bidirectional (Figure 1). Despite the degree of progress to date, unresolved issues remain. Patients and healthcare providers must be made aware that concomitant periodontal disease and COPD can worsen both conditions.

Older adults often have multiple chronic health problems [158]. In a super-aging society, health care for older adults is increasingly important [159]. Periodontal disease and COPD are common in the older population, and countermeasures against these diseases are crucial in rapidly aging countries [160,161]. Understanding the relationship between oral health and systemic conditions and assessing whether interventions for oral diseases can prevent systemic diseases (and vice versa) is crucial for effective health management. Investigating the multidisciplinary treatment approaches for a single disease may be important from the perspectives of extending healthy life expectancy, improving QOL, and minimizing healthcare costs. Further research is warranted on the relationship between these two diseases.

## Figures and Tables

**Table 2:** Studies investigating the association between periodontal disease and QOL in patients with COPD.

Author (Year)	Location	Study Design	Study Population	*n*	Measured Outcome	Main Findings
Zhou et al., 2011 [43]	China	Cross-sectional	Patients with COPD being treated at eight hospitals in Beijing	306	Periodontal parameters: PD, CAL, BOP, PI, and the number of missing teeth QOL measurement: SGRQ	The missing teeth were significantly associated with symptom score (*p* = 0.030) and activity score (*p* = 0.033); PI was also significantly associated with symptom score (*p* = 0.007).
Baldomero et al., 2019 [44]	USA	Case-control (exacerbators vs. non-exacerbators)	Individuals from the Minneapolis Veterans Affairs health care system	136 (patients with COPD: exacerbator, *n* = 70; non-exacerbator, *n* = 66)	Periodontal parameters: OHIP-5; PD, CAL, BOP, GI, PI, and caries risk assessment (subset of patients) QOL measurement: SGRQ	Worse OHRQoL as measured by OHIP-5 was associated with worse respiratory health scores (SGRQ total score): difficulty chewing (regression coefficient, 2.57; *p* = 0.023), painful ache in the mouth (regression coefficient, 5.43; *p* < 0.001), uncomfortable about appearance (regression coefficient, 3.17; *p* = 0.003), less flavor (regression coefficient, 3.53; *p* = 0.005), and difficulty performing jobs (regression coefficient, 7.31; *p* < 0.001).
Gaeckle et al., 2018 [45]	USA	Prospective cohort	Healthy individuals without lung disease and patients with severe COPD, recruited at a single medical center	30 (case, *n* = 20; control, *n* = 10)	Periodontal parameters: PI and OHIP-14 QOL measurement: electronic COPD daily diary	In patients with COPD, the number of teeth showed a significant positive correlation with the percentage of days with cough (*β* = 2.70, *p* = 0.04) and wheezing (*β* = 2.65, *p* = 0.01), whereas PI showed no significant correlation with daily respiratory symptoms.
Agado et al., 2012 [46]	USA	Randomized controlled trial	Patients diagnosed with COPD and chronic periodontitis	30 (magnetostrictive ultrasonic instrument, *n* = 10; hand instrument, *n* = 10; control, *n* = 10)	Periodontal parameters: PI and CAL QOL measurements: SGRQ-A and illness questionnaire (developed by the principal investigator)	SGRQ-A (symptom, *p* = 0.124; activity, *p* = 0.702; impact, *p* = 0.926) and illness questionnaire scores did not demonstrate significant differences in QOL or illness after periodontal debridement between groups.

BOP, bleeding on probing; CAL, clinical attachment loss; COPD, chronic obstructive pulmonary disease; GI, gingival index; OHIP, Oral Health Impact Profile; OHRQoL, oral health-related quality of life; PD, probing depth; PI, plaque index; QOL, quality of life; SGRQ, St. George’s Respiratory Questionnaire.

## Data Availability

No new data were created or analyzed in this study. Data sharing is not applicable to this article.
